# Overall and Telehealth Addiction Treatment Utilization by Age, Race, Ethnicity, and Socioeconomic Status in California After COVID-19 Policy Changes

**DOI:** 10.1001/jamahealthforum.2023.1018

**Published:** 2023-05-19

**Authors:** Vanessa A. Palzes, Felicia W. Chi, Verena E. Metz, Stacy Sterling, Asma Asyyed, Kathryn K. Ridout, Cynthia I. Campbell

**Affiliations:** 1 Center for Addiction and Mental Health Research, Division of Research, Kaiser Permanente Northern California, Oakland; 2 Department of Psychiatry and Behavioral Sciences, Weill Institute for Neurosciences, University of California, San Francisco; 3 Department of Health Systems Science, Kaiser Permanente Bernard J. Tyson School of Medicine, Pasadena, California; 4 Northern California Addiction Medicine and Recovery Services, The Permanente Medical Group, Inc, Santa Rosa; 5 The Permanente Medical Group, Inc, Santa Rosa, California

## Abstract

**Importance:**

Addiction treatment rapidly transitioned to a primarily telehealth modality (telephone and video) during the COVID-19 pandemic, raising concerns about disparities in utilization.

**Objective:**

To examine whether there were differences in overall and telehealth addiction treatment utilization after telehealth policy changes during the COVID-19 pandemic by age, race, ethnicity, and socioeconomic status.

**Design, Setting, and Participants:**

This cohort study examined electronic health record and claims data from Kaiser Permanente Northern California for adults (age ≥18 years) with drug use problems before the COVID-19 pandemic (from March 1, 2019, to December 31, 2019) and during the early phase of the COVID-19 pandemic (March 1, 2020, to December 31, 2020; hereafter referred to as COVID-19 onset). Analyses were conducted between March 2021 and March 2023.

**Exposure:**

The expansion of telehealth services during COVID-19 onset.

**Main Outcomes and Measures:**

Generalized estimating equation models were fit to compare addiction treatment utilization during COVID-19 onset with that before the COVID-19 pandemic. Utilization measures included the Healthcare Effectiveness Data and Information Set of treatment initiation and engagement (including inpatient, outpatient, and telehealth encounters or receipt of medication for opioid use disorder [OUD]), 12-week retention (days in treatment), and OUD pharmacotherapy retention. Telehealth treatment initiation and engagement were also examined. Differences in changes in utilization by age group, race, ethnicity, and socioeconomic status (SES) were examined.

**Results:**

Among the 19 648 participants in the pre–COVID-19 cohort (58.5% male; mean [SD] age, 41.0 [17.5] years), 1.6% were American Indian or Alaska Native; 7.5%, Asian or Pacific Islander; 14.3%, Black; 20.8%, Latino or Hispanic; 53.4%, White; and 2.5%, unknown race. Among the 16 959 participants in the COVID-19 onset cohort (56.5% male; mean [SD] age, 38.9 [16.3] years), 1.6% were American Indian or Alaska Native; 7.4%, Asian or Pacific Islander; 14.6%, Black; 22.2%, Latino or Hispanic; 51.0%, White; and 3.2%, unknown race. Odds of overall treatment initiation increased from before the COVID-19 pandemic to COVID-19 onset for all age, race, ethnicity, and SES subgroups except for patients aged 50 years or older; patients aged 18 to 34 years had the greatest increases (adjusted odds ratio [aOR], 1.31; 95% CI, 1.22-1.40). Odds of telehealth treatment initiation increased for all patient subgroups without variation by race, ethnicity, or SES, although increases were greater for patients aged 18 to 34 years (aOR, 7.17; 95% CI, 6.24-8.24). Odds of overall treatment engagement increased (aOR, 1.13; 95% CI, 1.03-1.24) without variation by patient subgroups. Retention increased by 1.4 days (95% CI, 0.6-2.2 days), and OUD pharmacotherapy retention did not change (adjusted mean difference, −5.2 days; 95% CI, −12.7 to 2.4 days).

**Conclusions:**

In this cohort study of insured adults with drug use problems, there were increases in overall and telehealth addiction treatment utilization after telehealth policies changed during the COVID-19 pandemic. There was no evidence that disparities were exacerbated, and younger adults may have particularly benefited from the transition to telehealth.

## Key Points

**Question:**

Were there addiction treatment utilization (overall and telehealth) differences by age, race, ethnicity, and socioeconomic status during the early phase of the COVID-19 pandemic after policies were enacted to expand telehealth?

**Findings:**

In this cohort study of 19 648 and 16 959 adults with drug use problems before and during the COVID-19 pandemic, respectively, during the COVID-19 pandemic, overall treatment initiation increased in all groups except patients aged 50 years or older and telehealth treatment initiation increased in all groups. Younger adults had greater increases in telehealth treatment initiation, but there was no significant variation in increases in overall and telehealth treatment initiation by race, ethnicity, or socioeconomic status.

**Meaning:**

Results of this study suggest that disparities in addiction treatment utilization were not exacerbated following expansion of telehealth during the early phase of the COVID-19 pandemic.

## Teaser

This cohort study examines whether there were overall and telehealth addiction treatment utilization differences by age, race, ethnicity, and socioeconomic status during the early phase of the COVID-19 pandemic.

## Introduction

The COVID-19 pandemic catalyzed a swift expansion of delivery of addiction treatment services via telehealth (telephone and video) facilitated by key changes in federal regulations. These changes included payment parity for telehealth and in-person visits, expanded Medicare coverage of telehealth services, and allowance of buprenorphine to be initially prescribed for opioid use disorder (OUD) treatment during telehealth visits without an in-person assessment.^1,2^ Accordingly, health care systems rapidly pivoted to provide telehealth services to follow disease mitigation guidelines and have fewer disruptions in care.^3,4^ These changes to broaden treatment access occurred at an important time,^5,6^ when drug use and drug overdose deaths were increasing.^7,8,9,10,11,12,13^

COVID-19–related telehealth policies at both the federal and the health care system level have largely remained in place. Studies of addiction treatment utilization after COVID-19 pandemic onset have shown mixed results. Some studies found initial decreases followed by increases that did not reach prepandemic levels,^14,15,16^ while other studies found overall increases^3^ or decreases.^16,17^ Studies have predominantly focused on expanded telehealth practices specifically for OUD treatment and reported mixed findings on treatment retention.^18,19,20^ Few studies have examined how addiction treatment utilization has changed following expansion of telehealth policies among all patients with drug use problems, not just those with OUD.

The shift to a primarily telehealth modality for specialty addiction treatment, which has traditionally consisted of intensive, in-person group therapy,^21^ has raised concerns about exacerbating disparities in treatment access and utilization. Prepandemic studies have found that Asian, Black, and Latino or Hispanic individuals with drug use problems were less likely to initiate treatment than were White individuals^22,23,24^ and that buprenorphine prescribing rates were lower among Black individuals with OUD compared with other racial and ethnic groups.^25^ Additionally, older and younger adults with drug use problems have been found to be less likely to initiate treatment than middle-aged adults.^23,24^ Limited evidence suggests that racial and ethnic disparities in addiction treatment utilization have persisted or worsened during the pandemic,^15,17,18^ while other studies of alcohol and behavioral health problems have not found increased disparities.^20,26,27^ While patients with limited technology access or skills (eg, patients with low income and older adults^28,29,30,31^) may have faced increased barriers to care during the pandemic, others may have found telehealth services to be more flexible and convenient, eliminating barriers such as transportation time and cost, the need for childcare, and stigma or anxiety related to attending in-person treatment.^32,33^

Greater understanding of changes in addiction treatment utilization during the COVID-19 pandemic among various subgroups of patients with drug use problems can help to tailor care as telehealth becomes more established in addiction treatment. The study goal was to examine changes in several overall and telehealth measures of addiction treatment utilization after telehealth policy changes during the first 10 months of the COVID-19 pandemic among adults with drug use problems, with a focus on differences by age group, race, ethnicity, and socioeconomic status (SES).

## Methods

In this cohort study, we examined electronic health records and claims data from Kaiser Permanente Northern California (KPNC) to compare trends in addiction treatment utilization during the early phase of the COVID-19 pandemic (March 1, 2020, to December 31, 2020; hereafter referred to as COVID-19 onset) with trends in the same months before the COVID-19 pandemic (March 1, 2019, to December 31, 2019). Analyses were conducted between March 2021 and March 2023. This study followed the Strengthening the Reporting of Observational Studies in Epidemiology (STROBE) reporting guideline for cohort studies.^34^ This minimal-risk study was approved by KPNC’s institutional review board with a waiver of informed consent based on 45 CFR §46.

### Setting

Kaiser Permanente Northern California is an integrated health care delivery system that serves more than 4.5 million members, approximately one-third of the Northern Californian population. The membership is racially and ethnically diverse and reflects the insured population of the US.^35^ The KPNC system offers addiction treatment in specialty clinics that patients can access without a referral or waiting lists and uses a group-based treatment model with case management and pharmacotherapy as needed. Methadone treatment is a covered benefit provided by referral to external opioid treatment programs.

Kaiser Permanente Northern California rapidly expanded its telehealth addiction care during the COVID-19 pandemic^3^ and implemented several strategies to help increase engagement, such as previsit calls by medical assistants to collect screening information and “tech-checks” to prepare patients for logging on to video visits. Although individual counseling sessions were available in person upon request, telehealth was the main modality of care delivery for outpatient services throughout the pandemic. Given the health care system’s familiarity with video technology, group therapy was adapted to video visits quickly after March 2020. Before the pandemic, telephone visits were available for individual appointments, although were used less.

### Sample

We identified adults (age ≥18 years) with drug use problems during the pre–COVID-19 and COVID-19 onset periods. To capture the full spectrum of patients with problematic drug use who might need treatment, drug use problems were defined as having either a drug use disorder (DUD) diagnosis (excluding tobacco use disorder) or an unhealthy substance use behavior diagnosis documented at an outpatient, inpatient, emergency department, or telehealth encounter or in a claim. Drug use disorder diagnoses were identified using *International Statistical Classification of Diseases and Related Health Problems, Tenth Revision* (*ICD-10*) diagnosis codes F11 to F16, F18, and F19 (excluding remission codes). Unhealthy substance use behavior diagnoses were identified using local diagnosis codes that standardize to *ICD-10* code Z72.89. A separate study by some of us^27^ examined alcohol use disorders (AUDs) but did not exclude patients if they had a comorbid diagnosis.

Index dates were defined as the first drug use problem diagnosis or inpatient discharge date (if the index diagnosis was made in an inpatient setting) during the pre–COVID-19 or COVID-19 onset period; patients could have an index date for each period. Patients were followed up to 6 months after the index date or until April 30, 2021 (study end for the COVID-19 onset cohort). To ensure sufficient time for identifying prior-year comorbid conditions using documented diagnoses, we excluded patients with less than 6 months of KPNC membership in the year before the index date. We also excluded patients without continuous membership or drug coverage during the 6 months after the index date, allowing a 30-day gap. Based on Healthcare Effectiveness Data and Information Set (HEDIS) specifications, we excluded patients who received a drug use problem diagnosis or pharmacotherapy for OUD in the 60 days before the index date.^36^

### Patient Characteristics and Exposure

From the electronic health records, we extracted patients’ sex, age at index, race (American Indian or Alaska Native, Asian or Pacific Islander, Black, White, and unknown race and ethnicity [American Indian or Alaska Native and unknown were combined into 1 category for analysis]), ethnicity (Latino or Hispanic), type of insurance, and type of index diagnosis (DUD or unhealthy substance use behavior). Race and ethnicity were patient-reported data supplemented with provider-reported and administrative sources. For patients with index DUD diagnoses, we also identified the type of DUD (patients could have >1). As a proxy measure of SES, we used the neighborhood deprivation index (NDI) from geocoded census-tract data from the 2019 American Community Survey and created a categorical variable based on quartiles of each cohort’s distribution, in which the first quartile reflected a lower NDI (higher SES) and the fourth quartile reflected a higher NDI (lower SES).^37^ We identified comorbid AUD and psychiatric disorder diagnoses (depression, bipolar disorder, anxiety disorder, obsessive-compulsive disorder, posttraumatic stress disorder, schizophrenia, schizoaffective disorder, and attention-deficit/hyperactivity disorder) based on *ICD-10* codes documented at encounters with the health care system in the year before the index date.^38^ As a measure of medical comorbidity burden, we calculated the Charlson Comorbidity Index score and created a categorical variable (0, 1-2, or ≥3).^39^ The primary exposure was an indicator for time (0, pre–COVID-19; 1, COVID-19 onset).

### Addiction Treatment Utilization Measures

#### Overall and Telehealth Treatment Initiation

Based on HEDIS specifications,^36^ overall treatment initiation was defined as initiating addiction treatment through an inpatient admission, outpatient visit, intensive outpatient encounter or partial hospitalization, or telehealth encounter (telephone, video, or unknown modality) or receipt of medication for OUD (dispensations of buprenorphine or oral naltrexone or a claim for methadone treatment) within 14 days of the index date. Telehealth treatment initiation was defined as initiating addiction treatment through a telehealth encounter or receipt of OUD medication within 14 days of the index date.

#### Overall and Telehealth Treatment Engagement

Also based on HEDIS specifications,^36^ overall treatment engagement was defined as having 2 or more additional addiction treatment–related services or OUD medication dispensations or claims within 34 days of the initiation visit or inpatient discharge date (if treatment was initiated through an inpatient admission). Telehealth treatment engagement was defined similarly but only included telehealth encounters and OUD medication dispensations or claims.

#### Treatment Retention

As a measure of time in treatment, retention was defined as the continuous number of days in outpatient addiction treatment (in-person or telehealth) in the 12 weeks following initiation until there was evidence that treatment had ended (ie, a 30-day gap). While there is no standard definition of retention, this approach aligned with previous research^40,41^ as well as the addiction treatment programs’ criteria for determining patient dropout.

#### OUD Pharmacotherapy Retention

Pharmacotherapy retention for OUD (buprenorphine and oral naltrexone) was defined as the continuous number of days treated in the 12 weeks after the initial prescription fill until a 30-day gap and was examined among patients with an index OUD diagnosis who initiated pharmacotherapy within 14 days of the index date. Methadone treatment was not included due to uncertainty about the days’ supply from administrative claims.

Addiction treatment engagement and retention measures were examined between 2 subgroups: patients who initiated any treatment and those who initiated treatment via telehealth. Analyses of engagement and retention were restricted to those who initiated treatment by December 6, 2019, for the pre–COVID-19 cohort or December 6, 2020, for the COVID-19 onset cohort to ensure that the pre–COVID-19 cohort had the full 12-week follow-up without overlap into the COVID-19 onset period.

### Statistical Analysis

All statistical analyses were conducted using SAS software, version 9.4 (SAS Institute Inc). We imputed missing NDI values (0.1%-0.3%) to the cohort’s mean.

For the pre–COVID-19 and COVID-19 onset cohorts of patients, we calculated unadjusted proportions and Wald 95% CIs for each of the addiction treatment utilization measures for all groups and by age group, race and ethnicity, and NDI quartile. We next fit a series of multivariable logistic generalized estimating equation models to compare treatment utilization in the COVID-19 onset period with that in the pre–COVID-19 period while accounting for within-participant correlation of repeated observations from the same patient. Each model included a time indicator and all patient characteristics (sex, age group, race, ethnicity, type of insurance, NDI quartile, index OUD diagnosis, Charlson Comorbidity Index score, any prior-year psychiatric disorder, prior-year AUD, and the index month). We examined potential disparities in pre–COVID-19 to COVID-19 onset changes in treatment utilization by age group, race, ethnicity, and NDI quartile. We included an interaction term between the time indicator and the relevant covariate individually in separate models that included all other covariates. If the interaction term (or joint test for multiple interaction terms) was significant at 2-sided *P* < .05, there was evidence that changes in utilization varied by subgroup. We then estimated the adjusted odds ratio (aOR) and 95% CI comparing treatment utilization during COVID-19 onset with that before the COVID-19 pandemic for each patient subgroup. We were unable to examine differences in changes in OUD pharmacotherapy retention by patient subgroups due to small sample sizes.

## Results

### Sample Characteristics

There were 19 648 patients in the pre–COVID-19 cohort and 16 959 patients in the COVID-19 onset cohort (eFigure in Supplement 1); there were 2434 patients (6.6%) in both cohorts. Among the participants in the pre–COVID-19 cohort, 41.5% were female, 58.5% were male, 1.6% were American Indian or Alaska Native, 7.5% were Asian or Pacific Islander, 14.3% were Black, 20.8% were Latino or Hispanic, 53.4% were White, and 2.5% had unknown race. Among the participants in the COVID-19 onset cohort, 43.5% were female, 56.5% were male, 1.6% were American Indian or Alaska Native, 7.4% were Asian or Pacific Islander, 14.6% were Black, 22.2% were Latino or Hispanic, 51.0% were White, and 3.2% had unknown race (Table). The mean (SD) age in the pre–COVID-19 cohort was 41.0 (17.5) years and in the COVID-19 onset cohort was 38.9 (16.3) years. Most patients had a DUD (68.3% and 69.7% in the pre–COVID-19 cohort and the COVID-19 onset cohort, respectively), with cannabis use disorder, OUD, and stimulant use disorder having the highest prevalence. In both the pre–COVID-19 cohort and the COVID-19 onset cohort, prior-year AUD (18.8% vs 19.1%) and psychiatric disorder diagnoses (59.1% vs 61.0%) were prevalent.

**Table.** Characteristics of Adults Identified With Drug Use Problems Before the COVID-19 Pandemic and During COVID-19 Onset in the Kaiser Permanente Northern California System^a^

Characteristic	Participants, No. (%)^b^	
	Pre–COVID-19 (n = 19 648)	COVID-19 onset (n = 16 959)
Sex		
Female	8150 (41.5)	7378 (43.5)
Male	11 498 (58.5)	9581 (56.5)
Age, y		
Mean (SD)	41.0 (17.5)	38.9 (16.3)
18-34	8769 (44.6)	8254 (48.7)
35-49	4442 (22.6)	4095 (24.1)
50-64	3939 (20.0)	3186 (18.8)
≥65	2498 (12.7)	1424 (8.4)
Race and ethnicity		
American Indian or Alaska Native	309 (1.6)	271 (1.6)
Asian or Pacific Islander^c^	1481 (7.5)	1261 (7.4)
Black	2803 (14.3)	2470 (14.6)
Latino or Hispanic	4082 (20.8)	3764 (22.2)
White	10 484 (53.4)	8644 (51.0)
Unknown^d^	489 (2.5)	549 (3.2)
Type of insurance		
Medicaid	2508 (12.8)	2697 (15.9)
Medicare	3512 (17.9)	2005 (11.8)
Commercial	13 577 (69.1)	12 206 (72.0)
Other or unknown	51 (0.3)	51 (0.3)
Neighborhood deprivation index quartile^e^		
1	5471 (27.8)	4765 (28.1)
2	5397 (27.5)	4502 (26.5)
3	4894 (24.9)	4227 (24.9)
4	3848 (19.6)	3426 (20.2)
Unknown	38 (0.2)	39 (0.2)
Index drug diagnosis		
Unhealthy substance use behavior	6238 (31.7)	5144 (30.3)
Drug use disorder^f^		
Any	13 410 (68.3)	11 815 (69.7)
Cannabis	7059 (35.9)	6596 (38.9)
Cocaine	1021 (5.2)	790 (4.7)
Opioid	2537 (12.9)	1984 (11.7)
Polysubstance	1125 (5.7)	956 (5.6)
Sedative, anxiolytic, or hypnotic	482 (2.5)	376 (2.2)
Stimulant	1814 (9.2)	1618 (9.5)
Other^g^	1834 (9.3)	1583 (9.3)
Charlson Comorbidity Index score		
0	12 203 (62.1)	10 724 (63.2)
1-2	4780 (24.3)	4232 (25.0)
≥3	2665 (13.6)	2003 (11.8)
Prior-year alcohol use disorder diagnosis	3688 (18.8)	3241 (19.1)
Prior-year psychiatric disorder diagnosis^f^		
Any	11 606 (59.1)	10 340 (61.0)
Anxiety disorder	7772 (39.6)	7083 (41.8)
Depression	7340 (37.4)	6489 (38.3)
Index month		
March	2429 (12.4)	2045 (12.1)
April	2402 (12.2)	1678 (9.9)
May	2266 (11.5)	1976 (11.7)
June	1949 (9.9)	2078 (12.3)
July	2050 (10.4)	1769 (10.4)
August	1879 (9.6)	1548 (9.1)
September	1773 (9.0)	1564 (9.2)
October	1879 (9.6)	1629 (9.6)
November	1514 (7.7)	1415 (8.3)
December	1507 (7.7)	1257 (7.4)

^a^ The pre–COVID-19 period was from March 1, 2019, to December 31, 2019, and COVID-19 onset represents the initial period of the pandemic from March 1, 2020, to December 31, 2020.

^b^ Percentages may not add to 100% because of rounding.

^c^ In the pre–COVID-19 cohort, there were 1350 (6.9%) Asian patients (Chinese, Filipino, Japanese, Korean, South Asian, Other Southeast Asian, Vietnamese, or other or unknown Asian patients), 128 (0.7%) Pacific Islander patients, and 3 (<0.1%) Asian/Pacific Islander patients. In the COVID-19 onset cohort, there were 1156 (6.8%) Asian patients, 103 (0.6%) Pacific Islander patients, and 2 (<0.1%) Asian/Pacific Islander patients.

^d^ Patients with unknown race did not specify any 1 category.

^e^ Quartiles range from 1 to 4, with 1 indicating the lowest neighborhood deprivation index (higher socioeconomic status), and 4 indicating the highest neighborhood deprivation index (lower socioeconomic status).

^f^ Patients could have more than 1 diagnosis at their index episode; thus, percentages may not add to 100%.

^g^ Other includes hallucinogens, inhalants, and other drugs.

### Addiction Treatment Utilization and Overall Treatment Initiation

**Figure 1.**
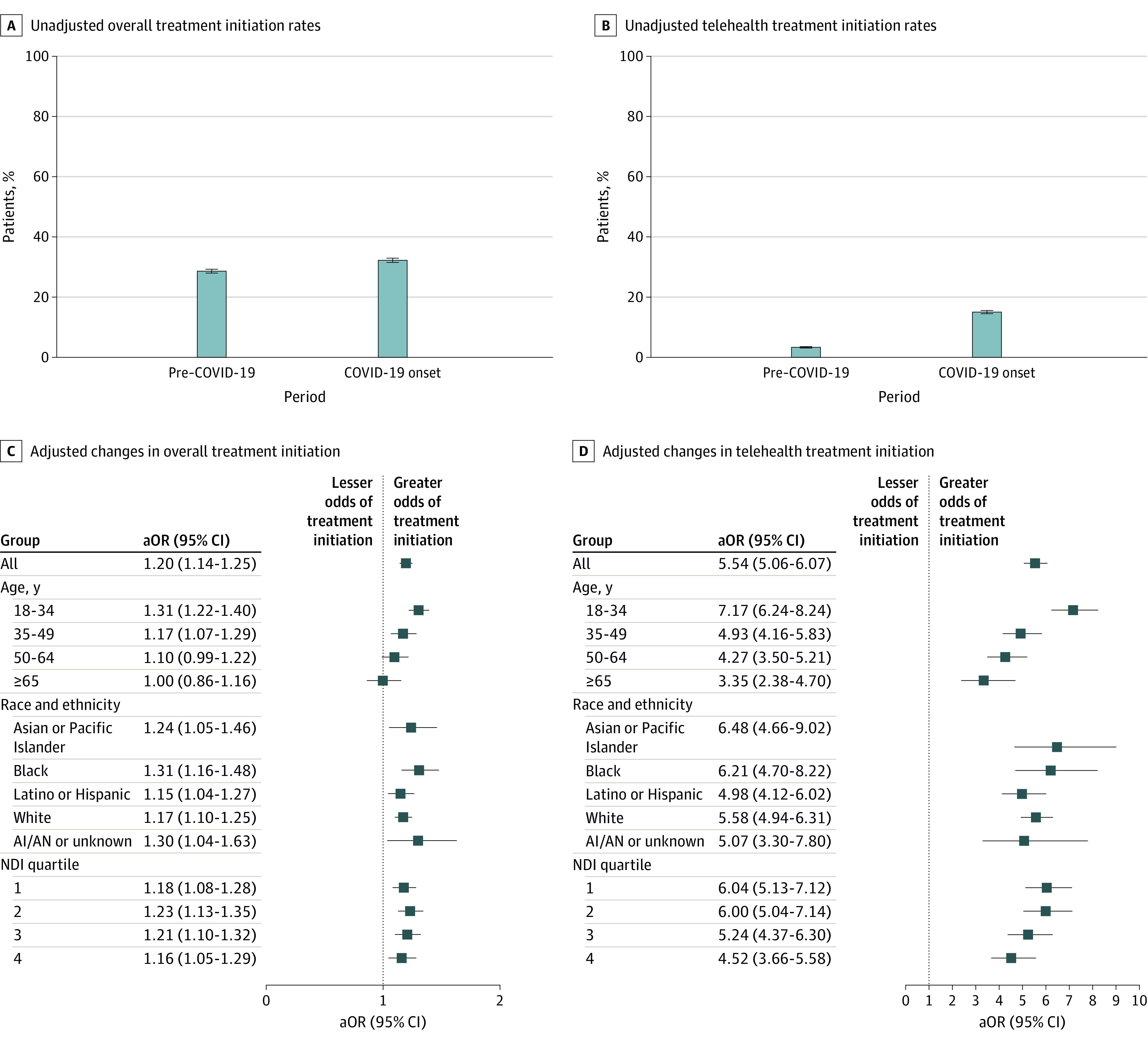
Unadjusted Overall and Telehealth Addiction Treatment Initiation Rates and Adjusted Changes Over Time Between Pre–COVID-19 and the Time of COVID-19 Onset The pre–COVID-19 period was from March 1, 2019, to December 31, 2019, and COVID-19 onset represents the initial period of the pandemic from March 1, 2020, to December 31, 2020. Error bars represent 95% CIs. C and D, Neighborhood deprivation index (NDI) quartiles range from 1 to 4, with 1 indicating the lowest NDI (higher socioeconomic status) and 4 indicating the highest NDI (lower socioeconomic status). Markers indicate adjusted odds ratios (aORs), with horizontal lines indicating 95% CIs. AI/AN indicates American Indian or Alaska Native.

Unadjusted and adjusted addiction treatment utilization measures are given in eTables 1 to 6 in Supplement 1. The unadjusted proportions of patients who initiated any treatment were 28.6% (95% CI, 28.0%-29.2%) before the COVID-19 pandemic and 32.2% (95% CI, 31.5%-32.9%) during COVID-19 onset (Figure 1A and eTable 1 in Supplement 1). After adjusting for differences in patient characteristics across time, the odds of overall treatment initiation were higher during the COVID-19 onset period compared with before the COVID-19 pandemic (aOR, 1.20; 95% CI, 1.14-1.25) (Figure 1C). The increase was observed for patients aged 18 to 34 years (aOR, 1.31; 95% CI, 1.22-1.40) and those aged 35 to 49 years (aOR, 1.17; 95% CI, 1.07-1.29) but not for those aged 50 years or older. Overall treatment initiation increased, with no significant variation for all race and ethnicity groups and NDI quartiles.

#### Telehealth Treatment Initiation

The unadjusted proportions of patients who initiated telehealth treatment increased from 3.3% (95% CI, 3.1%-3.6%) before the COVID-19 pandemic to 15.0% (95% CI, 14.5%-15.5%) during COVID-19 onset (Figure 1B and eTable 1 in Supplement 1). Multivariable analyses revealed a greater odds of telehealth treatment initiation during COVID-19 onset compared with before the COVID-19 pandemic (aOR, 5.54; 95% CI, 5.06-6.07) (Figure 1D). Odds of telehealth treatment initiation increased for all age, race, ethnicity, and NDI groups, but the magnitude of increases significantly differed by age group. Patients aged 18 to 34 years had the greatest increase (aOR, 7.17; 95% CI, 6.24-8.24).

#### Overall Treatment Engagement

**Figure 2.**
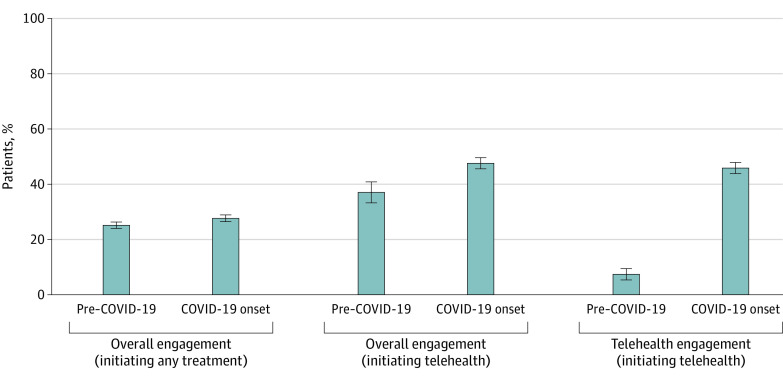
Unadjusted Overall and Telehealth Addiction Treatment Engagement Rates The pre–COVID-19 period was from March 1, 2019, to December 31, 2019, and COVID-19 onset represents the initial period of the pandemic from March 1, 2020, to December 31, 2020. Error bars represent 95% CIs. Among patients who initiated any treatment, odds of treatment engagement were higher during COVID-19 onset compared with before the COVID-19 pandemic (adjusted odds ratio [aOR], 1.13; 95% CI, 1.03-1.24). Among patients who initiated telehealth treatment, odds of treatment engagement (aOR, 1.70; 95% CI, 1.41-2.06) and telehealth treatment engagement (aOR, 11.76; 95% CI, 8.60-16.09) were higher during COVID-19 onset compared with before the pandemic.

Among patients who initiated any treatment before the COVID-19 pandemic (n = 5621) and during COVID-19 onset (n = 5463), the unadjusted proportions who engaged in any treatment were 25.2% (95% CI, 24.0%-26.3%) and 27.7% (95% CI, 26.4%-28.9%), respectively (Figure 2 and eTable 2 in Supplement 1). Multivariable analyses suggested higher odds of overall treatment engagement during COVID-19 onset compared with before the COVID-19 pandemic (aOR, 1.13; 95% CI, 1.03-1.24) and no significant variation by patient characteristics.

Among patients who initiated telehealth treatment before the COVID-19 pandemic (n = 621) and during COVID-19 onset (n = 2360), the unadjusted proportions who engaged in any treatment were 37.0% (95% CI, 33.2%-40.8%) and 47.6% (95% CI, 45.6%-49.6%), respectively (Figure 2 and eTable 3 in Supplement 1). In multivariable analyses, the odds of overall treatment engagement increased from before the COVID-19 pandemic to COVID-19 onset (aOR, 1.70; 95% CI, 1.41-2.06) with no significant variation by patient characteristics.

#### Telehealth Treatment Engagement

The unadjusted proportion of patients who engaged in telehealth treatment in the pre–COVID-19 cohort (n = 621) was 7.4% (95% CI, 5.4%-9.5%) and in the COVID-19 onset cohort (n = 2360) was 45.9% (95% CI, 43.8%-47.9%) (Figure 2 and eTable 3 in Supplement 1). Odds of telehealth treatment engagement were higher during COVID-19 onset compared with before the COVID-19 pandemic (aOR, 11.76; 95% CI, 8.60-16.09). There was no significant variation in telehealth treatment engagement by age, race, ethnicity, or NDI quartile.

#### Treatment and OUD Pharmacotherapy Retention

**Figure 3.**
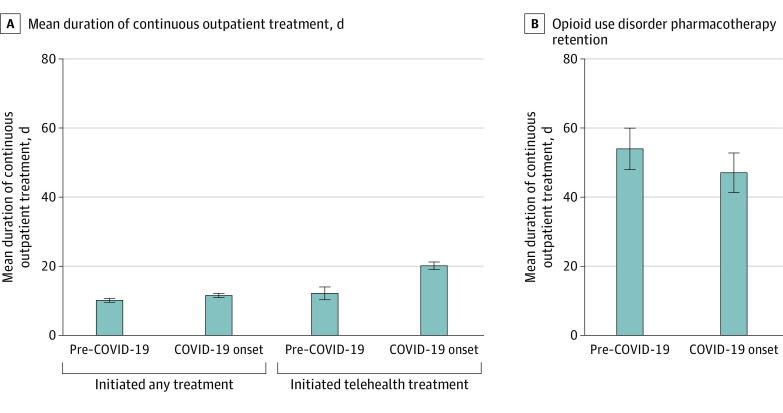
Unadjusted Addiction Treatment Retention Rate The pre–COVID-19 period was from March 1, 2019, to December 31, 2019, and COVID-19 onset represents the initial period of the pandemic from March 1, 2020, to December 31, 2020. Error bars represent 95% CIs. A, Adjusted mean differences (aMDs) comparing retention during COVID-19 onset with the pre-COVID-19 period were 1.4 days (95% CI, 0.6-2.2 days) among patients who initiated any treatment and 7.9 days (95% CI, 5.7-10.0 days) among those who initiated treatment using telehealth. B, Opioid use disorder pharmacotherapy retention was examined among patients with index diagnoses who received a dispensation of buprenorphine or naltrexone within 14 days (aMD, −5.2 days; 95% CI, −12.7 to 2.4 days).

Treatment retention increased by 1.4 days (95% CI, 0.6-2.2 days) among patients who initiated any treatment and 7.9 days (95% CI, 5.7-10.0 days) among those who initiated telehealth treatment (Figure 3A), with no significant variation by patient characteristics (eTables 4 and 5 in Supplement 1). There were no significant changes in the mean number of continuous days treated with buprenorphine or oral naltrexone from before the COVID-19 pandemic to COVID-19 onset (adjusted mean difference, −5.2 days; 95% CI, −12.7 to 2.4 days) (Figure 3B), and there was no significant variation by patient characteristics (eTable 6 in Supplement 1).

## Discussion

In this large, racially and ethnically diverse cohort study of insured adults with drug use problems, rates of initiation and engagement and duration of retention in addiction treatment were higher during the first 10 months of the COVID-19 pandemic compared with the same months before the pandemic, especially for telehealth services. While increases in overall addiction treatment utilization may have been modest, it is notable that disparities in utilization by age, race, ethnicity, and SES were not exacerbated in this health care system. The findings may be generalizable to other health care systems with similarly insured populations with drug use problems and may have important policy implications given that telehealth is likely to remain a prominent modality of care delivery. This study contributes to the literature by examining several measures of addiction treatment utilization not studied previously, to our knowledge, and by evaluating differences by the key patient characteristics of age, race, ethnicity, and SES.

The increases observed across the study treatment utilization measures may have been associated with several mechanisms. First, overall increases might have been explained by increases in telehealth utilization, which may reflect broadened access to treatment due to the flexibility and convenience of telehealth care.^42,43^ Although telehealth use was likely motivated by pandemic-related federal and health care system policies, patient preferences, which we could not measure in this study, may have also been involved. Prior studies showed that telehealth may be a satisfactory treatment modality for treatment-seeking patients with drug use problems^44,45^ and may be associated with improved treatment retention.^46^ Patients may have found telehealth treatment to be more comfortable than in-person care, without barriers such as transportation and stigma (eg, fear of being negatively perceived if seen in treatment).^32,33,47^ Additionally, the pandemic disrupted daily routines (eg, employment loss, more time at home), so patients may have had more time and space to initiate treatment. Finally, increases in treatment utilization may also be associated with increased patient need and higher demand for addiction treatment services. Several studies suggest that substance use problems increased during the pandemic.^12,13,48^

This study identified age differences in changes in treatment utilization from before the COVID-19 pandemic to COVID-19 onset. Younger patients were more likely to initiate overall addiction treatment during COVID-19 onset compared with before the COVID-19 pandemic, while older patients were not. These differences seem to be explained by less telehealth use by older patients, consistent with prior studies that suggest a digital divide by age,^49,50,51^ but could also signify that the transition to telehealth may have helped to attract younger patients, who have shown low treatment initiation rates in prepandemic studies.^23,24^ Similar age differences have been reported in studies examining telehealth treatment for other conditions in this health care system^27^ and other settings.^52^ The health care system’s strategies to support telehealth use (eg, previsit calls and tech-checks) may have helped to prevent an increasing digital divide for older adults. Yet, older patients may need even greater support and a longer time to learn how to interface digitally.

We found no significant differences in the magnitude of increases in overall and telehealth treatment utilization by race, ethnicity, or SES, despite the potential for treatment disparities to widen following pandemic-related changes in care delivery. Asian or Pacific Islander, Black, and Latino or Hispanic patients have had historically lower addiction treatment utilization rates compared with White patients,^22,23,24^ and there has been evidence of worsening treatment disparities during the pandemic in other settings.^15,17^ It is possible that previously discussed mechanisms associated with increases in telehealth use (eg, flexibility, lessened stigma) also have contributed to mitigating potential disparities by race and ethnicity. This study’s sample was stably insured, which made it more possible to disentangle associations of race, ethnicity, and SES from insurance status and may explain why the findings differ from those of other studies. It is important to note that the unadjusted telehealth treatment initiation rate for Black patients remained lower than for White patients in our study, reinforcing that disparities still exist even if gaps did not worsen.

There were no changes in OUD pharmacotherapy retention, consistent with a recent study of adult Medicare beneficiaries that found no changes in the proportion of eligible days covered with buprenorphine or injectable naltrexone.^18^ Pharmacotherapy retention did not decrease, which supports permanent adoption of the pandemic-related regulations that broadened telehealth and flexibility of OUD medication prescribing.^48^ Yet, the lack of increased retention, even in an insured system after policies broadened access to buprenorphine treatment, reinforces the continued challenge of retaining patients in OUD treatment.

### Limitations

Our study has limitations. It was conducted among a stably insured population in an integrated health care delivery system; thus, the findings may not generalize to the overall US population without similar levels of insurance and access to care. The risk of insurance loss during the pandemic may have been heightened.^53^ While this study examined the first 10 months of the COVID-19 pandemic (with follow-up extending to April 2021), it is important for future studies to examine treatment beyond this time frame, as the pandemic has continued to impact health care delivery. We were unable to examine other select external addiction resources (eg, self-help and 12-step programs, Narcotics Anonymous, and Alcoholics Anonymous) but included external services for which there were claims filed within the health care system (eg, methadone treatment). However, we did not include methadone in our OUD pharmacotherapy retention measure because we were unable to determine days’ supply, which may explain why we did not find increases in OUD medication retention, as a recent study observed.^18^ While the NDI is a multidimensional construct of neighborhood SES, we used it as a proxy of individual SES. While residual confounding by individual SES is possible, we expect it to be minimal given that KPNC covers largely urban and suburban areas, which are less prone to discordance between individual and neighborhood SES.^54^ Finally, our study did not examine outcomes of telehealth treatment utilization (eg, DUD remission), which will be important to examine in a future study.

## Conclusions

In this cohort study of an insured population with drug use problems from a large, integrated health care system, addiction treatment utilization, especially telehealth, increased during the COVID-19 pandemic compared with before the pandemic. Patients aged 18 to 34 years had a greater likelihood of telehealth treatment initiation and may have benefited from the transition to telehealth in this health care system. Importantly, disparities by race, ethnicity, and SES did not worsen during the early phase of the COVID-19 pandemic. Further research is needed to understand how telehealth can complement in-person care for diverse patient populations.

## Notes

Supplement 1. **eFigure.** Cohort Flow Diagram of Study Inclusion and Exclusion Criteria **eTable 1.** Unadjusted Proportions of Patients Who Initiated Addiction Treatment and Adjusted Odds Ratios (aORs) Comparing Treatment Initiation During COVID-19 Onset With Pre–COVID-19 **eTable 2.** Unadjusted Proportions of Patients Who Engaged in Addiction Treatment and Adjusted Odds Ratios (aORs) Comparing Treatment Engagement During COVID-19 Onset With Pre–COVID-19, Among Patients Who Initiated Any Treatment **eTable 3.** Unadjusted Proportions of Patients Who Engaged in Addiction Treatment and Adjusted Odds Ratios (aORs) Comparing Treatment Engagement During COVID-19 Onset With Pre–COVID-19, Among Patients Who Initiated Treatment via Telehealth **eTable 4.** Unadjusted Continuous Mean Days in Outpatient Addiction Treatment and Adjusted Mean Differences (aMDs) Comparing Treatment Retention During COVID-19 Onset With Pre–COVID-19, Among Patients Who Initiated Any Treatment **eTable 5.** Unadjusted Continuous Mean Days in Outpatient Addiction Treatment and Adjusted Mean Differences (aMDs) Comparing Treatment Retention During COVID-19 Onset With Pre–COVID-19, Among Patients Who Initiated Treatment via Telehealth **eTable 6.** Unadjusted Continuous Mean Days of Treatment With Opioid Use Disorder (OUD) Medications and Adjusted Mean Differences (aMDs) Comparing OUD Pharmacotherapy Retention During COVID-19 Onset With Pre–COVID-19, Among Patients With OUD Who Initiated Pharmacotherapy

Supplement 2.
Data Sharing Statement
