# Supplementary material for: Overall and Telehealth Addiction Treatment Utilization by Age, Race, Ethnicity, and Socioeconomic Status in California After COVID-19 Policy Changes
Source: JAMA Health Forum. 2023 May 19;4(5):e231018. doi: 10.1001/jamahealthforum.2023.1018 (PMC10199344; doi:10.1001/jamahealthforum.2023.1018)
Supplement: Supplement 1. — eFigure. Cohort Flow Diagram of Study Inclusion and Exclusion Criteria eTable 1. Unadjusted Proportions of Patients Who Initiated Addiction Treatment and Adjusted Odds Ratios (aORs) Comparing Treatment Initiation During COVID-19 Onset With Pre–COVID-19 eTable 2. Unadjusted Proportions of Patients Who Engaged in Addiction Treatment and Adjusted Odds Ratios (aORs) Comparing Treatment Engagement During COVID-19 Onset With Pre–COVID-19, Among Patients Who Initiated Any Treatment eTable 3. Unadjusted Proportions of Patients Who Engaged in Addiction Treatment and Adjusted Odds Ratios (aORs) Comparing Treatment Engagement During COVID-19 Onset With Pre–COVID-19, Among Patients Who Initiated Treatment via Telehealth eTable 4. Unadjusted Continuous Mean Days in Outpatient Addiction Treatment and Adjusted Mean Differences (aMDs) Comparing Treatment Retention During COVID-19 Onset With Pre–COVID-19, Among Patients Who Initiated Any Treatment eTable 5. Unadjusted Continuous Mean Days in Outpatient Addiction Treatment and Adjusted Mean Differences (aMDs) Comparing Treatment Retention During COVID-19 Onset With Pre–COVID-19, Among Patients Who Initiated Treatment via Telehealth eTable 6. Unadjusted Continuous Mean Days of Treatment With Opioid Use Disorder (OUD) Medications and Adjusted Mean Differences (aMDs) Comparing OUD Pharmacotherapy Retention During COVID-19 Onset With Pre–COVID-19, Among Patients With OUD Who Initiated Pharmacotherapy [file jamahealthforum-e231018-s001.pdf]

Supplemental Online Content

Palzes VA, Chi FW, Metz VE, et al. Overall and telehealth addiction treatment utilization by age, race, ethnicity, and socioeconomic status in California after COVID-19 policy changes. *JAMA Health Forum*. 2023;4(5):e231018. doi:10.1001/jamahealthforum.2023.1018

eFigure. Cohort Flow Diagram of Study Inclusion and Exclusion Criteria
eTable 1. Unadjusted Proportions of Patients Who Initiated Addiction Treatment and Adjusted Odds Ratios (aORs) Comparing Treatment Initiation During COVID-19 Onset With Pre–COVID-19
eTable 2. Unadjusted Proportions of Patients Who Engaged in Addiction Treatment and Adjusted Odds Ratios (aORs) Comparing Treatment Engagement During COVID-19 Onset With Pre–COVID-19, Among Patients Who Initiated Any Treatment
eTable 3. Unadjusted Proportions of Patients Who Engaged in Addiction Treatment and Adjusted Odds Ratios (aORs) Comparing Treatment Engagement During COVID-19 Onset With Pre–COVID-19, Among Patients Who Initiated Treatment via Telehealth
eTable 4. Unadjusted Continuous Mean Days in Outpatient Addiction Treatment and Adjusted Mean Differences (aMDs) Comparing Treatment Retention During COVID-19 Onset With Pre–COVID-19, Among Patients Who Initiated Any Treatment
eTable 5. Unadjusted Continuous Mean Days in Outpatient Addiction Treatment and Adjusted Mean Differences (aMDs) Comparing Treatment Retention During COVID-19 Onset With Pre–COVID-19, Among Patients Who Initiated Treatment via Telehealth
eTable 6. Unadjusted Continuous Mean Days of Treatment With Opioid Use Disorder (OUD) Medications and Adjusted Mean Differences (aMDs) Comparing OUD Pharmacotherapy Retention During COVID-19 Onset With Pre–COVID-19, Among Patients With OUD Who Initiated Pharmacotherapy

This supplemental material has been provided by the authors to give readers additional information about their work.

© 2023 Palzes VA et al. *JAMA Health Forum*.

eFigure. Cohort Flow Diagram of Study Inclusion and Exclusion Criteria

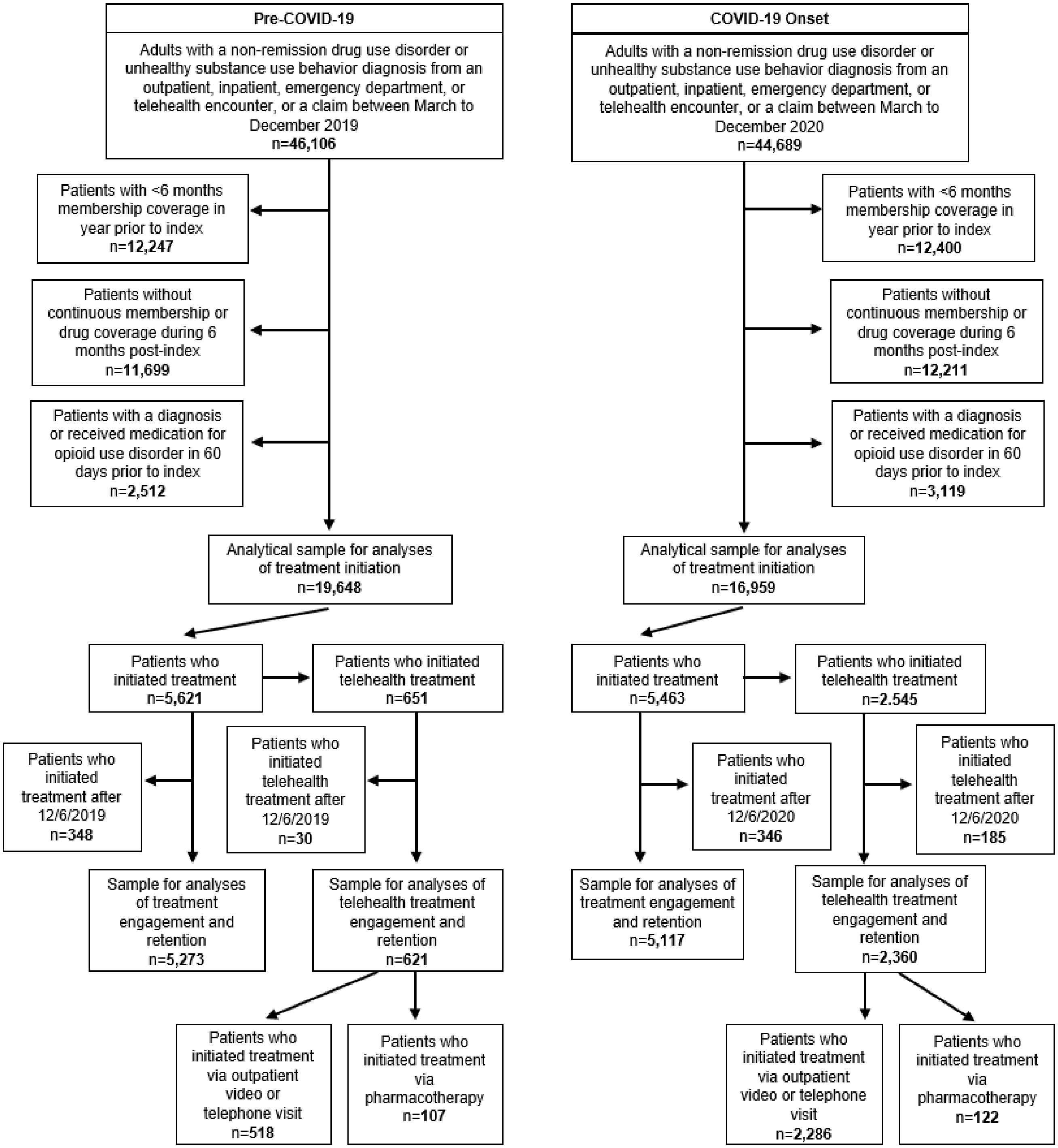

© 2023 Palzes VA et al. *JAMA Health Forum*.

eTable 1. Unadjusted Proportions of Patients Who Initiated Addiction Treatment and Adjusted Odds Ratios (aORs) Comparing Treatment Initiation During COVID-19 Onset to Pre–COVID-19

	Unadjusted % (95% CI)				
**Outcome**	**Pre-COVID-19 (n=19,648)**	**COVID-19 Onset (n=16,959)**	**COVID-19 Onset vs Pre-COVID-19, aOR (95% CI)[a]**	**p-value[b]**	**Interaction p-value[c]**
**Overall treatment initiation[d]**					
All groups	28.6 (28.0, 29.2)	32.2 (31.5, 32.9)	1.20 (1.14, 1.25)	<.001	-
By age (y)					0.002
18-34	26.3 (25.4, 27.3)	31.9 (30.9, 32.9)	1.31 (1.22, 1.40)	<.001	
35-49	28.6 (27.2, 29.9)	31.8 (30.3, 33.2)	1.17 (1.07, 1.29)	<.001	
50-64	31.3 (29.8, 32.7)	33.4 (31.7, 35.0)	1.10 (0.99, 1.22)	0.08	
≥65	32.5 (30.6, 34.3)	32.6 (30.2, 35.0)	1.00 (0.86, 1.16)	0.99	
By race and ethnicity					0.43
API	28.2 (25.9, 30.5)	33.4 (30.8, 36.0)	1.24 (1.05, 1.46)	0.01	
Black	26.7 (25.1, 28.4)	31.9 (30.1, 33.7)	1.31 (1.16, 1.48)	<.001	
Latino or Hispanic	28.8 (27.4, 30.2)	31.7 (30.2, 33.2)	1.15 (1.04, 1.27)	0.005	
White	29.5 (28.6, 30.3)	32.6 (31.7, 33.6)	1.17 (1.10, 1.25)	<.001	
AI/AN or unknown	23.9 (21.0, 26.9)	29.3 (26.2, 32.4)	1.30 (1.04, 1.63)	0.02	
By NDI quartile					0.81
1	30.0 (28.8, 31.2)	33.3 (32.0, 34.6)	1.18 (1.08, 1.28)	<.001	
2	28.1 (26.9, 29.3)	32.4 (31.1, 33.8)	1.23 (1.13, 1.35)	<.001	
3	28.0 (26.7, 29.2)	32.0 (30.6, 33.4)	1.21 (1.10, 1.32)	<.001	
4	28.1 (26.7, 29.6)	30.7 (29.2, 32.3)	1.16 (1.05, 1.29)	0.005	
**Telehealth treatment initiation[e]**					
All groups	3.3 (3.1, 3.6)	15.0 (14.5, 15.5)	5.54 (5.06, 6.07)	<.001	-
By age (y)					<.001
18-34	2.9 (2.6, 3.3)	16.3 (15.6, 17.1)	7.17 (6.24, 8.24)	<.001	
35-49	4.3 (3.8, 4.9)	16.7 (15.6, 17.9)	4.93 (4.16, 5.83)	<.001	
50-64	3.7 (3.1, 4.2)	13.0 (11.8, 14.1)	4.27 (3.50, 5.21)	<.001	
≥65	2.3 (1.7, 2.9)	6.9 (5.6, 8.2)	3.35 (2.38, 4.70)	<.001	
By race and ethnicity					0.57
API	3.2 (2.3, 4.1)	16.7 (14.6, 18.7)	6.48 (4.66, 9.02)	<.001	
Black	2.2 (1.7, 2.8)	11.6 (10.4, 12.9)	6.21 (4.70, 8.22)	<.001	
Latino or Hispanic	3.7 (3.1, 4.3)	15.2 (14.0, 16.3)	4.98 (4.12, 6.02)	<.001	
White	3.5 (3.1, 3.8)	15.7 (14.9, 16.5)	5.58 (4.94, 6.31)	<.001	
AI/AN or unknown	3.4 (2.1, 4.6)	14.8 (12.3, 17.2)	5.07 (3.30, 7.80)	<.001	
By NDI quartile					0.14
1	3.6 (3.1, 4.1)	17.2 (16.1, 18.3)	6.04 (5.13, 7.12)	<.001	
2	3.2 (2.8, 3.7)	15.8 (14.7, 16.8)	6.00 (5.04, 7.14)	<.001	
3	3.2 (2.7, 3.7)	14.1 (13.0, 15.1)	5.24 (4.37, 6.30)	<.001	
4	3.1 (2.6, 3.7)	12.1 (11.0, 13.2)	4.52 (3.66, 5.58)	<.001	

AI/AN=American Indian or Alaska Native; API=Asian or Pacific Islander; NDI=neighborhood deprivation index.

[a] Adjusted odds ratios (aOR) and 95% CIs represent the change in odds of the treatment utilization measure, comparing the COVID-19 onset period to the pre-COVID-19 period, for all groups or the specific subgroup, adjusting for sex, age group, race, ethnicity, type of insurance, NDI quartile, index opioid use disorder diagnosis, Charlson comorbidity index, any prior-year psychiatric disorder, prior-year alcohol use disorder, and index month.

[b] The p-value for the aOR.

[c] The p-value for the interaction term or the joint test of multiple interaction terms.

[d] Of adults in the pre-COVID-19 and COVID-19 onset cohorts who initiated any treatment, 48.7% and 47.1% were identified in an inpatient setting at index, 1.4% and 1.5% went to inpatient treatment, 39.0% and 5.8% had an in-person outpatient visit, 9.6% and 45.1% had a telehealth encounter, and 1.3% and 1.6% received medication for OUD, respectively.

[e] Of adults in the pre-COVID-19 and COVID-19 onset cohorts who initiated telehealth treatment, 81.4% and 48.0% initiated via telephone visits, 1.4% and 48.5% initiated via video visits, 9.8% and 1.1% initiated via unknown telehealth modality, and 7.4% and 2.4% initiated via OUD pharmacotherapy, respectively

© 2023 Palzes VA et al. *JAMA Health Forum*.

eTable 2. Unadjusted Proportions of Patients Who Engaged in Addiction Treatment and Adjusted Odds Ratios (aORs) Comparing Treatment Engagement During COVID-19 Onset With Pre–COVID-19, Among Patients Who Initiated Any Treatment

	**Unadjusted % (95% CI)**				
**Overall treatment engagement**	**Pre-COVID-19 (n=5,621)**	**COVID-19 Onset (n=5,463)**	**COVID-19 Onset vs Pre-COVID-19, aOR (95% CI)[a]**	**p-value[b]**	**Interaction p-value[c]**
All groups	25.2 (24.0, 26.3)	27.7 (26.4, 28.9)	1.13 (1.03, 1.24)	0.01	-
By age (y)					0.13
18-34	28.9 (27.0, 30.8)	30.5 (28.7, 32.3)	1.16 (1.02, 1.33)	0.02	
35-49	31.6 (29.0, 34.3)	31.7 (29.1, 34.3)	1.02 (0.86, 1.23)	0.80	
50-64	21.6 (19.2, 23.9)	22.1 (19.5, 24.7)	1.05 (0.84, 1.30)	0.69	
≥65	9.6 (7.5, 11.7)	13.6 (10.4, 16.8)	1.65 (1.14, 2.39)	0.008	
By race and ethnicity					0.65
API	29.4 (24.9, 34.0)	29.0 (24.5, 33.5)	1.00 (0.73, 1.38)	0.99	
Black	19.9 (16.9, 22.8)	21.6 (18.7, 24.6)	1.07 (0.82, 1.41)	0.60	
Latino or Hispanic	24.2 (21.7, 26.7)	28.6 (25.9, 31.2)	1.27 (1.05, 1.55)	0.02	
White	26.2 (24.6, 27.8)	28.6 (26.9, 30.3)	1.10 (0.97, 1.24)	0.15	
AI/AN or unknown	25.5 (19.2, 31.8)	29.3 (23.2, 35.4)	1.24 (0.78, 1.98)	0.36	
By NDI quartile					0.92
1	26.9 (24.7, 29.1)	29.5 (27.2, 31.8)	1.12 (0.95, 1.32)	0.19	
2	26.2 (23.9, 28.5)	28.4 (26.0, 30.8)	1.10 (0.92, 1.31)	0.30	
3	24.6 (22.3, 27.0)	27.9 (25.4, 30.3)	1.19 (0.99, 1.44)	0.06	
4	21.8 (19.2, 24.3)	23.6 (20.9, 26.3)	1.10 (0.88, 1.38)	0.38	

AI/AN=American Indian or Alaska Native; API=Asian or Pacific Islander, NDI=neighborhood deprivation index.

[a] Adjusted odds ratios (aOR) and 95% CIs represent the change in odds of the treatment utilization measure, comparing the COVID-19 onset period to the pre-COVID-19 period, for all groups or the specific subgroup, adjusting for sex, age group, race, ethnicity, type of insurance, NDI quartile, index opioid use disorder diagnosis, Charlson comorbidity index, any prior-year psychiatric disorder, prior-year alcohol use disorder, and index month.

[b] The p-value for the aOR.

[c] The p-value for the interaction term or the joint test of multiple interaction terms.

© 2023 Palzes VA et al. *JAMA Health Forum*.

eTable 3. Unadjusted Proportions of Patients Who Engaged in Addiction Treatment and Adjusted Odds Ratios (aORs) Comparing Treatment Engagement During COVID-19 Onset With Pre–COVID-19, Among Patients Who Initiated Treatment via Telehealth

Outcome	Unadjusted % (95% CI) Pre-COVID-19 (n=621)	COVID-19 Onset (n=2,360)	COVID-19 Onset vs Pre-COVID-19, aOR (95% CI)[a]	p-value[b]	Interaction p-value[c]
**Overall treatment engagement**					
All groups	37.0 (33.2, 40.8)	47.6 (45.6, 49.6)	1.70 (1.41, 2.06)	<.001	-
By age (y)					0.67
18-34	33.3 (27.4, 39.2)	46.2 (43.4, 49.0)	1.92 (1.43, 2.57)	<.001	
35-49	42.5 (35.3, 49.7)	52.9 (49.0, 56.8)	1.66 (1.18, 2.32)	0.003	
50-64	37.9 (29.8, 45.9)	44.6 (39.7, 49.6)	1.40 (0.93, 2.11)	0.11	
≥65	33.3 (20.8, 45.9)	43.2 (33.2, 53.1)	1.71 (0.84, 3.48)	0.14	
By race and ethnicity					0.95
API	35.6 (21.6, 49.5)	46.6 (39.6, 53.7)	1.76 (0.91, 3.40)	0.09	
Black	40.4 (27.6, 53.1)	44.7 (38.8, 50.7)	1.34 (0.73, 2.45)	0.35	
Latino or Hispanic	34.0 (26.3, 41.8)	46.8 (42.5, 51.0)	1.76 (1.19, 2.60)	0.004	
White	38.3 (33.2, 43.4)	48.7 (45.9, 51.4)	1.73 (1.34, 2.22)	<.001	
AI/AN or unknown	32.0 (13.7, 50.3)	47.6 (37.9, 57.2)	1.91 (0.75, 4.84)	0.17	
By NDI quartile					0.40
1	39.9 (32.9, 46.9)	46.6 (43.1, 50.1)	1.44 (1.04, 2.00)	0.03	
2	38.7 (31.3, 46.1)	48.5 (44.7, 52.3)	1.71 (1.19, 2.44)	0.004	
3	38.6 (30.9, 46.3)	49.6 (45.5, 53.8)	1.68 (1.16, 2.43)	0.006	
4	27.7 (19.4, 36.0)	45.0 (40.0, 50.0)	2.38 (1.47, 3.85)	<.001	
**Telehealth treatment engagement**					
All groups	7.4 (5.4, 9.5)	45.9 (43.8, 47.9)	11.76 (8.60, 16.09)	<.001	-
By age (y)					0.10
18-34	5.3 (2.5, 8.1)	44.2 (41.4, 47.0)	15.35 (8.60, 27.38)	<.001	
35-49	6.6 (3.0, 10.3)	52.1 (48.2, 56.0)	16.45 (9.02, 29.97)	<.001	
50-64	9.3 (4.5, 14.1)	43.3 (38.4, 48.3)	8.35 (4.49, 15.53)	<.001	
≥65	14.8 (5.3, 24.3)	36.8 (27.1, 46.5)	3.97 (1.65, 9.53)	0.002	
By race and ethnicity					0.90
API	6.7 (0.0, 14.0)	46.1 (39.1, 53.2)	13.41 (3.95, 45.50)	<.001	
Black	5.3 (0.0, 11.1)	42.5 (36.5, 48.4)	14.76 (4.55, 47.86)	<.001	
Latino or Hispanic	5.6 (1.8, 9.3)	44.7 (40.5, 49.0)	14.24 (6.71, 30.25)	<.001	
White	8.3 (5.4, 11.2)	46.9 (44.2, 49.7)	11.00 (7.39, 16.37)	<.001	
AI/AN or unknown	12.0 (0.0, 24.7)	46.6 (37.0, 56.2)	7.08 (1.92, 26.10)	0.003	
By NDI quartile					0.97
1	6.9 (3.3, 10.5)	44.9 (41.4, 48.4)	12.68 (6.98, 23.05)	<.001	
2	8.3 (4.2, 12.5)	46.2 (42.4, 50.0)	10.84 (6.12, 19.21)	<.001	
3	7.8 (3.6, 12.1)	48.6 (44.4, 52.7)	11.18 (6.18, 20.21)	<.001	
4	6.3 (1.8, 10.7)	43.2 (38.2, 48.2)	12.97 (5.66, 29.71)	<.001	

AI/AN=American Indian or Alaska Native; API=Asian or Pacific Islander, NDI=neighborhood deprivation index.
[a] Adjusted odds ratios (aOR) and 95% confidence intervals (CI) represent the change in odds of the treatment utilization measure, comparing the COVID-19 onset period to the pre-COVID-19 period, for all groups or the specific subgroup, adjusting for sex, age group, race, ethnicity, type of insurance, NDI quartile, index opioid use disorder diagnosis, Charlson comorbidity index, any prior-year psychiatric disorder, prior-year alcohol use disorder, and index month.
[b] The p-value for the aOR.
[c] The p-value for the interaction term or the joint test of multiple interaction terms.

© 2023 Palzes VA et al. *JAMA Health Forum*.

eTable 4. Unadjusted Continuous Mean Days in Outpatient Addiction Treatment and Adjusted Mean Differences (aMDs) Comparing Treatment Retention During COVID-19 Onset With Pre–COVID-19, Among Patients Who Initiated Any Treatment

	Unadjusted Mean Days (95% CI)				
**Treatment retention**	**Pre-COVID-19 (n=5,621)**	**COVID-19 Onset (n=5,463)**	**COVID-19 Onset vs Pre-COVID-19, aMD (95% CI)[a]**	**p-value[b]**	**Interaction p-value[c]**
All groups	10.1 (9.6, 10.7)	11.5 (10.9, 12.1)	1.4 (0.6, 2.2)	<.001	-
By age (y)					0.22
18-34	11.3 (10.4, 12.1)	11.9 (11.1, 12.8)	1.2 (0, 2.4)	0.05	
35-49	12.6 (11.3, 13.8)	13.8 (12.4, 15.1)	1.4 (-0.4, 3.2)	0.12	
50-64	9.2 (8.0, 10.4)	9.8 (8.5, 11.1)	0.8 (-1.0, 2.5)	0.39	
≥65	4.4 (3.3, 5.5)	7.1 (5.2, 9.0)	3.5 (1.4, 5.6)	0.001	
By race and ethnicity					0.42
API	11.1 (9.1, 13.1)	12.2 (10.0, 14.3)	1.4 (-1.4, 4.3)	0.33	
Black	8.1 (6.7, 9.6)	8.5 (7.1, 9.8)	0.4 (-1.6, 2.3)	0.71	
Latino or Hispanic	10.2 (9.0, 11.5)	11.0 (9.7, 12.3)	0.8 (-0.9, 2.5)	0.34	
White	10.5 (9.7, 11.2)	12.3 (11.4, 13.2)	1.7 (0.5, 2.8)	0.004	
AI/AN or unknown	9.2 (6.4, 12.0)	13.6 (10.3, 16.9)	4.5 (0.4, 8.6)	0.03	
By NDI quartile					0.097
1	10.7 (9.7, 11.8)	12.8 (11.6, 13.9)	2.1 (0.6, 3.6)	0.007	
2	10.9 (9.8, 12.0)	11.7 (10.5, 12.9)	0.7 (-0.8, 2.3)	0.36	
3	9.6 (8.5, 10.7)	12.0 (10.8, 13.3)	2.5 (0.9, 4.1)	0.003	
4	8.7 (7.5, 9.9)	8.7 (7.5, 9.9)	0 (-1.7, 1.5)	0.91	

AI/AN=American Indian or Alaska Native; API=Asian or Pacific Islander, NDI=neighborhood deprivation index.
[a] Adjusted mean differences (aMD) and 95% confidence intervals (CI) represent the change in mean days of treatment retention, comparing the COVID-19 onset period to the pre-COVID-19 period, for all groups or the specific subgroup, adjusting for sex, age group, race, ethnicity, type of insurance, NDI quartile, index opioid use disorder diagnosis, Charlson comorbidity index, any prior-year psychiatric disorder, prior-year alcohol use disorder, and index month.
[b] The p-value for the aOR.
[c] The p-value for the interaction term or the joint test of multiple interaction terms.

© 2023 Palzes VA et al. *JAMA Health Forum*.

eTable 5. Unadjusted Continuous Mean Days in Outpatient Addiction Treatment and Adjusted Mean Differences (aMDs) Comparing Treatment Retention During COVID-19 Onset With Pre–COVID-19, Among Patients Who Initiated Treatment via Telehealth

	Unadjusted Mean Days (95% CI)				
**Treatment retention**	**Pre-COVID-19 (n=518)**	**COVID-19 Onset (n=2,286)**	**COVID-19 Onset vs Pre-COVID-19, aMD (95% CI)[a]**	**p-value[b]**	**Interaction p-value[c]**
All groups	12.2 (10.3, 14.0)	20.1 (19.0, 21.2)	7.9 (5.7, 10.0)	<.001	-
By age (y)					0.59
18-34	11.2 (8.4, 14.0)	18.5 (17.1, 20.0)	7.2 (4.2, 10.3)	<.001	
35-49	14.3 (10.4, 18.2)	23.2 (21.0, 25.4)	8.5 (4.1, 12.9)	<.001	
50-64	12.3 (8.4, 16.3)	19.7 (17.0, 22.4)	6.8 (2.1, 11.4)	0.004	
≥65	9.2 (3.9, 14.6)	22.2 (15.9, 28.4)	12.8 (4.9, 20.7)	0.001	
By race and ethnicity					0.07
API	16.8 (9.0, 24.6)	19.3 (15.7, 22.9)	3.7 (-4.4, 11.8)	0.37	
Black	6.6 (3.1, 10.1)	18.7 (15.6, 21.8)	11.9 (7.2, 16.6)	<.001	
Latino or Hispanic	14.1 (9.8, 18.3)	18.1 (15.9, 20.3)	3.7 (-0.9, 8.4)	0.12	
White	11.8 (9.4, 14.2)	21.2 (19.6, 22.7)	9.4 (6.6, 12.3)	<.001	
AI/AN or unknown	7.9 (-0.2, 15.9)	23.3 (17.5, 29.1)	12.7 (3.7, 21.8)	0.006	
By NDI quartile					0.52
1	13.6 (10.1, 17.1)	20.5 (18.6, 22.4)	7.3 (3.4, 11.1)	<.001	
2	12.9 (9.3, 16.5)	20.5 (18.3, 22.6)	7.9 (3.7, 12.1)	<.001	
3	10.5 (7.0, 14.0)	21.6 (19.2, 23.9)	10.4 (6.2, 14.5)	<.001	
4	10.7 (6.2, 15.1)	16.7 (14.2, 19.1)	5.6 (0.8, 10.5)	0.023	

AI/AN=American Indian or Alaska Native; API=Asian or Pacific Islander, NDI=neighborhood deprivation index.
[a] Adjusted mean differences (aMD) and 95% confidence intervals (CI) represent the change in mean days of treatment retention, comparing the COVID-19 onset period to the pre-COVID-19 period, for all groups or the specific subgroup, adjusting for sex, age group, race, ethnicity, type of insurance, NDI quartile, index opioid use disorder diagnosis, Charlson comorbidity index, any prior-year psychiatric disorder, prior-year alcohol use disorder, and index month.
[b] The p-value for the aOR.
[c] The p-value for the interaction term or the joint test of multiple interaction terms.

© 2023 Palzes VA et al. *JAMA Health Forum*.

eTable 6. Unadjusted Continuous Mean Days of Treatment With Opioid Use Disorder (OUD) Medications and Adjusted Mean Differences (aMDs) Comparing OUD Pharmacotherapy Retention During COVID-19 Onset With Pre–COVID-19, Among Patients With OUD Who Initiated Pharmacotherapy

	Unadjusted Mean Days (95% CI)				
**OUD pharmacotherapy**	**Pre-COVID-19 (n=107)**	**COVID-19 Onset (n=122)**	**COVID-19 Onset vs Pre-COVID-19, aMD (95% CI)[a]**	**p-value[b]**	**Interaction p-value[c]**
All groups	54.0 (48.0, 60.0)	47.0 (41.3, 52.8)	-5.2 (-12.7, 2.4)	0.18	-

[a] Adjusted mean difference (aMD) and 95% confidence interval (CI) represent the change in mean days of treatment retention, comparing the COVID-19 onset period to the pre-COVID-19 period for all groups, adjusting for sex, age group, race, ethnicity, type of insurance, NDI quartile, index opioid use disorder diagnosis, Charlson comorbidity index, any prior-year psychiatric disorder, prior-year alcohol use disorder, and index month.
[b] The p-value for the aOR.
[c] The p-value for the interaction term or the joint test of multiple interaction terms.

© 2023 Palzes VA et al. *JAMA Health Forum*.
