# Supplementary material for: Overall and Telehealth Addiction Treatment Utilization by Age, Race, Ethnicity, and Socioeconomic Status in California After COVID-19 Policy Changes
Source: JAMA Health Forum. 2023 May 19;4(5):e231018. doi: 10.1001/jamahealthforum.2023.1018 (PMC10199344; doi:10.1001/jamahealthforum.2023.1018)
Supplement: Supplement 2. — Data Sharing Statement [file jamahealthforum-e231018-s002.pdf]

Data Sharing Statement

Palzes. Overall and Telehealth Addiction Treatment Utilization by Age, Race, Ethnicity, and Socioeconomic Status in California After COVID-19 Policy Changes. *JAMA Health Forum*. Published May 19, 2023. doi:10.1001/jamahealthforum.2023.1018

Data
Data available: Yes
Data types: Other (please specify)
Additional Information: The analytical datasets from this project consist of aggregate level data that comply with Kaiser Permanente Northern California (KPNC) electronic medical record and administrative and clinical database policies per HIPAA regulations. External investigators may contact the corresponding author to initiate a request for study data to support new study proposals or manuscripts. Approval of requests will evaluate whether the proposed project is of high scientific merit and consistent with KPNC policies per HIPAA regulations. Approved requests will need to consider data sharing agreements which Kaiser Permanente has with NIH. Data will be de-identified prior to secure file transmission; however, researchers who seek access to individual level data will be required to execute a data sharing agreement (DSA) prior to release for sharing. The DSA would require: (1) a commitment to using the data for the approved project only while maintaining confidentiality of individual participants and ensuring there is no disclosure of Kaiser Permanente proprietary information; (2) a commitment to protecting the data using appropriate secure computer technology; (3) a commitment to destroying or returning the data after approved project analyses are completed; and (4) a commitment to meet any requirements that might be stipulated by the Institutional Review Board at KPNC.
How to access data: Please send requests for data to Vanessa.A.Palzes@kp.org and Cynthia.I.Campbell@kp.org.
When available: With publication

Supporting Documents
Document types: None

Additional Information
Who can access the data: External investigators may contact the corresponding author to initiate a request for study data to support new study proposals or manuscripts.
Types of analyses: Data analyses must be intended for study proposals or manuscripts. Approval of data requests will evaluate whether the proposed project is of high scientific merit and consistent with KPNC policies per HIPAA regulations.
Mechanisms of data availability: Approved requests will need to consider data sharing agreements which Kaiser Permanente has with NIH. Data will be de-identified prior to secure file transmission; however, researchers who seek access to individual level data will be required to execute a data sharing agreement (DSA) prior to release for sharing. The DSA would require: (1) a commitment to using the data for the approved project only while maintaining confidentiality of individual participants and ensuring there is no disclosure of Kaiser Permanente proprietary information; (2) a commitment to protecting the data using appropriate secure computer technology; (3) a commitment to destroying or returning the data after approved project analyses are completed; and (4) a commitment to meet any requirements that might be stipulated by the Institutional Review Board at KPNC.
